# Causality between genetically predicted type 2 diabetes and ankle fracture risk: A 2-sample Mendelian randomization study

**DOI:** 10.1097/MD.0000000000047504

**Published:** 2026-02-06

**Authors:** Wenren Wu, Zhiqing Chen, Binshan Zhang, Linfeng Luo

**Affiliations:** a Orthopedics Department, Dongguan Hospital of Guangzhou University of Chinese Medicine, Dong Guan, Guangdong Province, China.

**Keywords:** ankle joint, causal relationship, diabetes mellitus, fractures

## Abstract

It has been proven that diabetes mellitus plays an important role in the occurrence and development of joint fractures. In this study, a 2-sample Mendelian randomization (MR) analysis was conducted to investigate the causal relationship between diabetes and ankle fractures. We pooled the data from the published genome-wide association studies. Diabetes mellitus type 2 was derived from pooled genome-wide association study data of 655,666 European individuals (61,714 patients and 1178 controls). Data on ankle fractures were derived from pooled genome-wide association study data in a total of 460,340 European individuals (6479 patients and 453,861 controls). Using diabetes-associated loci as instrumental variables, we used inverse variance weighting, MR-Egger, weighted median, simple multivariate analysis and weighted multivariate analysis to evaluate the association between diabetes and ankle fracture risk. Reverse MR analysis was performed on the Diabetes mellitus type 2 that were found to be causally associated with ankle fractures in forward MR analysis. Sensitivity analysis was used to evaluate the robustness of the results. Statistical analysis showed a significant causal relationship between diabetes and ankle fractures (inverse variance weighting: OR = 1.07, 95% CI = 1.01–1.32, *P* = .02). Diabetes mellitus is associated with an increased risk of ankle fracture. The results of MR analysis can be used as a guide for the screening of diabetes and ankle fractures, which is helpful to improve the awareness of screening, early diagnosis and early treatment.

## 1. Introduction

The number of people with diabetes has reached 537 million worldwide, and the growth trend is still unchecked. Globally, an estimated 12.1% of deaths in 2021 were associated with diabetes; diabetes and its complications cause US $966 billion in global economic losses.^[1]^ In China, the prevalence of diabetes has also shown a rapid increase. According to the latest epidemiological survey data, the prevalence of diabetes in China is as high as 12.4%, but the blood glucose control rate is still <50%.^[2]^ Type 2 diabetes accounts for nearly 90% of the estimated 537 million diabetes cases worldwide. The number of people affected is increasing rapidly, with worrying trends for children and young people. Early detection and proactive management are essential to prevent and reduce the burden of microvascular and macrovascular complications and death.^[3]^ Ankle fracture is a common intra-articular fracture. The classic ankle fracture entails a loss of stability in the ankle mortise, whereas the Pilon fracture involves catastrophic destruction of the distal tibial articular surface; the former is primarily induced by rotational forces, while the latter results from high-energy axial loading.^[4]^ Because the structure of ankle joint is complex and it is a weight-bearing joint, studies have shown that patients with diabetes mellitus type 2 (T2DM) often have some soft tissue damage.^[5]^ However, whether there is a causal relationship between T2DM and ankle fracture needs further study.

Mendelian randomization (MR) studies are a method to assess the causal relationship between various exposures and disease outcomes, which utilizes genetic variants as instrumental variables (IV).^[6]^ This analysis makes causal inference by using genetic variants as modifiable risk factors or proxies for health outcomes.^[7]^ Since genes are randomly assigned from conception, genetic variation is largely independent of other factors.^[8]^ MR Studies need to meet 3 basic assumptions: association hypothesis: genetic variants are associated with risk factors; independence assumption: genetic variation is not associated with any known or unknown confounding factors; exclusion restriction hypothesis: genetic variation affects outcomes only through risk factors.^[9]^

Two-sample MR involves 2 different study populations. For example, the data of T2DM is measured in 1 sample, and the data of ankle fractures is measured in another sample. This design has 2 advantages: first, not all MR Studies are required to collect risk factors and outcomes. Second, the results can be very large (typically >50,000), allowing the use of pooled results from genome-wide association studies and thus making the results precise and statistically efficient. At the same time, it can also solve the problem of sample collection difficulty or high cost.^[10]^

In this study, we used MR approach to explore the potential causal relationship between genetic variants associated with ankle fractures and T2DM.

## 2. Materials and methods

### 2.1. Information

T2DM was derived from pooled genome-wide association study (GWAS) data of 655,666 European individuals, including 61,714 patients and 1178 controls, with 5,030,727 single nucleotide polymorphisms (SNPs). Summary data from https://gwas.mrcieu.ac.uk/datasets/ebi-a-GCST006867/ in public. Data on ankle fractures were derived from pooled GWAS data in a total of 460,340 European individuals, with 6479 patients with ankle fractures and 453,861 controls. The pooled GWAS results included 9,851,867 SNPs. https://gwas.mrcieu.ac.uk/datasets/ukb-b-15582/, all participants are European.

No additional ethical approval was required because the data used in this MR Study were in a publicly available database. The exposure factors used in this study, T2DM, and outcome ankle fractures sample information are detailed in Table 1.

**Table 1:** Details of samples included in MR study.

Variable	Sample size (n)	Case group (n)	Control group (n)	Sample origin	Yr of publication	URL
Diabetes mellitus type 2	655,666	61,714	1178	Europe	2018	https://gwas.mrcieu.ac.uk/datasets/ebi-a-GCST006867/
Ankle fractures	460,340	6479	453,861	Europe	2018	https://gwas.mrcieu.ac.uk/datasets/ukb-b-15582/

### 2.2. Methods

#### 2.2.1. Study design

**Figure 1.**
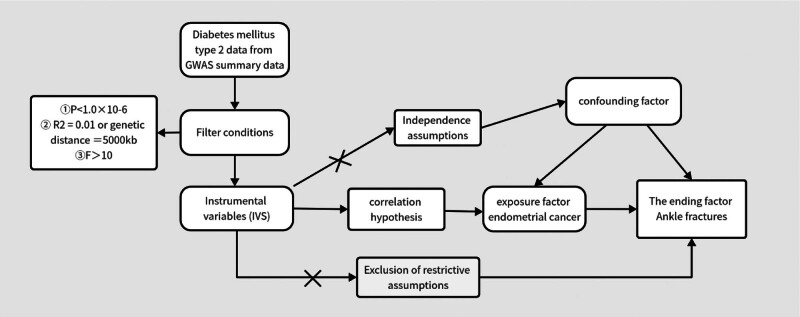
Schematic of MR core assumptions. GWAS = genome-wide association study, MR = Mendelian randomization.

This study used a 2-sample MR approach, with T2DM as the exposure factor, SNPs as the IV, and ankle fractures as the outcome factor, to verify the causal relationship between the gene-predicted exposure factor T2DM and the outcome ankle fractures. MR analysis needs to follow 3 core assumptions^[11]^: The assumption of association requires that the SNPs used as IVs must be closely related to the exposure factor of interest. The assumption of independence assumes that the selected genetic variant is independent of other confounders that may affect exposure and outcome. The exclusivity assumption prescribes that the effect of SNPs on the outcome variable can only be achieved through this exposure factor and not through any other route (see Fig. 1).

#### 2.2.2. Selection of instrumental variables

SNPs were required to meet the following criteria: the significance threshold of the SNPs at the genome-wide level must be <1.0 × 10^−6^, indicating their significant association with endometrial cancer; the degree of explained variation (*R*^2^) of the SNPs must be at least 0.01 or the genetic distance must not exceed 5000 kb; Those SNPs that had *R*^2^ values >0.01 with the most significant SNPs within a distance of 5000 kb were removed, that is, SNPs in linkage disequilibrium were excluded. SNPs with palindromic structures that could affect the accuracy of the analysis were excluded. SNPs with an *F*-statistic <10 were removed to filter out potentially weak IVs. Weak IVs are those genetic variants that, despite their association with the exposure under study, have weak explanatory power for the exposure. Because of their small effect on the exposure factor, these variants are almost useless in providing the statistical power needed to test the research hypothesis, which can lead to impaired precision in causal effect estimates. Problems that may be caused by weak IVs include increasing the risk of false positive errors (i.e., type I errors).^[9]^ The strength of IVs can be assessed by the *F*-statistic, which for a single SNP can be calculated by the following formula: *F* = bate^2^/se^2,[12]^ which is less affected by weak instrumental bias when *F* > 10. Based on the GWAS results of T2DM and ankle fractures, we can collate SNPs with the same alleles in order to match the effect size of exposure and outcome.^[13]^

#### 2.2.3. Statistical treatment

In this study, the inverse variance weighting (IVW) method was used as the main study method to evaluate the potential causal association between T2DM and the risk of ankle fractures. This method is most applicable when genotype and exposure factors are highly balanced and can produce unbiased estimates. In this study, the intercept test in MR-Egger’s method was used to detect whether there was horizontal pleiotropy bias in IVs. If *P* > .05, there was no horizontal pleiotropy.^[14]^ In addition, a leave-one-out analysis was performed to assess the impact of individual SNPs on the significant effect. The Cochran’s *Q* test was used to test for heterogeneity among IVs. When the *P* value is <.05, it indicates that there is significant heterogeneity, and the IVW of the random effect model is recommended for analysis.^[15]^ For sensitivity analysis, the leave-one-out method was used, that is, after removing each SNPs in turn, the IVW method was used again to estimate the causal effect of the remaining SNPs. The aim of this method is to judge the reliability of these estimation results by assessing the possible influence of some specific effect-level genetic variants (SNPs) on the causal inference results of MR. However, when there is multiplicity or imbalance in the data, the IVW method may lead to bias. Therefore, as complementary means, the MR-Egger method, weighted median method (WME), simple mode method, and weighted mode method were used for the analysis.

All the above analyses were performed in R software environment (version 4.3.3; Guangzhou, Guangdong, China), and statistical analysis and data visualization were completed with the help of the “2 SampleMR” R package (version 0.5.6). Since IVW method is more efficient than the other 4 MR methods in detecting causal effect, this study chooses IVW method for causal effect testing. Due to the relaxation of the threshold requirement, to avoid increasing the risk of type I error, and at the same time verify whether the study results are affected by multiple testing.

## 3. Results

### 3.1. Determination of instrumental variables

The R software was used to select SNPs associated with T2DM with genome-wide significance (*P* < .05) for pooling. After excluding the control step of linkage disequilibrium interference, a total of 116 SNPs were included as IV. As a single SNP of IV, the *F*-statistic ranges from 29.94 to 1578.20, indicating that there is a strong correlation between SNPs and exposure, and the analysis results of MR are unlikely to be biased by weak IVs (see Table S1, Supplemental Digital Content, https://links.lww.com/MD/R298).

### 3.2. Causal relationship between T2DM and ankle fractures

**Figure 2.**
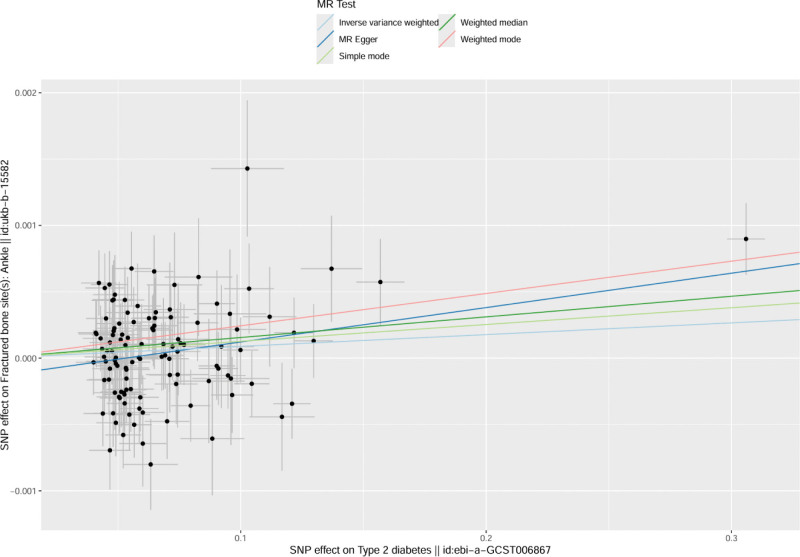
Scatter plot of MR analysis results for both samples (Notes: The overall trend was positive. With the onset of type 2 diabetes, the risk of fracture gradually increased). MR = Mendelian randomization.

The MR analysis indicated that the direction of causality across the 5 methods was consistent, suggesting that T2DM may increase the risk of ankle fractures. However, statistical significance was only observed with the IVW method (OR = 1.07, 95% CI = 1.01–1.32, *P* = .02), indicating a 7% increased risk. The other methods (MR Egger, WME, simple mode, and weighted mode) did not reach statistical significance, as their confidence intervals included 1 and their *P*-values were >.05. Thus, the evidence of a causal relationship is stronger when using the IVW method but less conclusive with the others (*P* < .05, see Table 2 and Fig. 2).

**Table 2:** Results of MR analysis of the relationship between T2DM and ankle fracture risk.

Exposure factors	Method	*OR*	95% *CI*	*P*
T2DM	MR-Egger	1.21	0.97–1.51	.01
	WME	1.05	0.97–1.31	.01
	IVW	1.07	1.01–1.32	.03
	Simple mode	0.98	0.83–1.15	.39
	Weighted mode	0.98	0.83–1.15	.01

CI = confidence interval, IVW = inverse variance weighting, MR = Mendelian randomization, OR = odds ratio, T2DM = diabetes mellitus type 2, WME = weighted median.

### 3.3. Results of heterogeneity test and pleiotropy test

Cochran’s *Q* test indicated that a *P*-value of <.01 suggests significant heterogeneity among the IVs. The heterogeneity result for IVW was *P* = .07, indicating no significant heterogeneity among the 116 SNPs when using this method. The heterogeneity result for MR-Egger regression was *P* = .01, suggesting borderline significant heterogeneity among the SNPs. The intercept *P* of MR-Egger regression analysis was >0.05, which indicated that there was a potential confounding bias in the analysis results.

### 3.4. Sensitivity analysis

**Figure 3.**
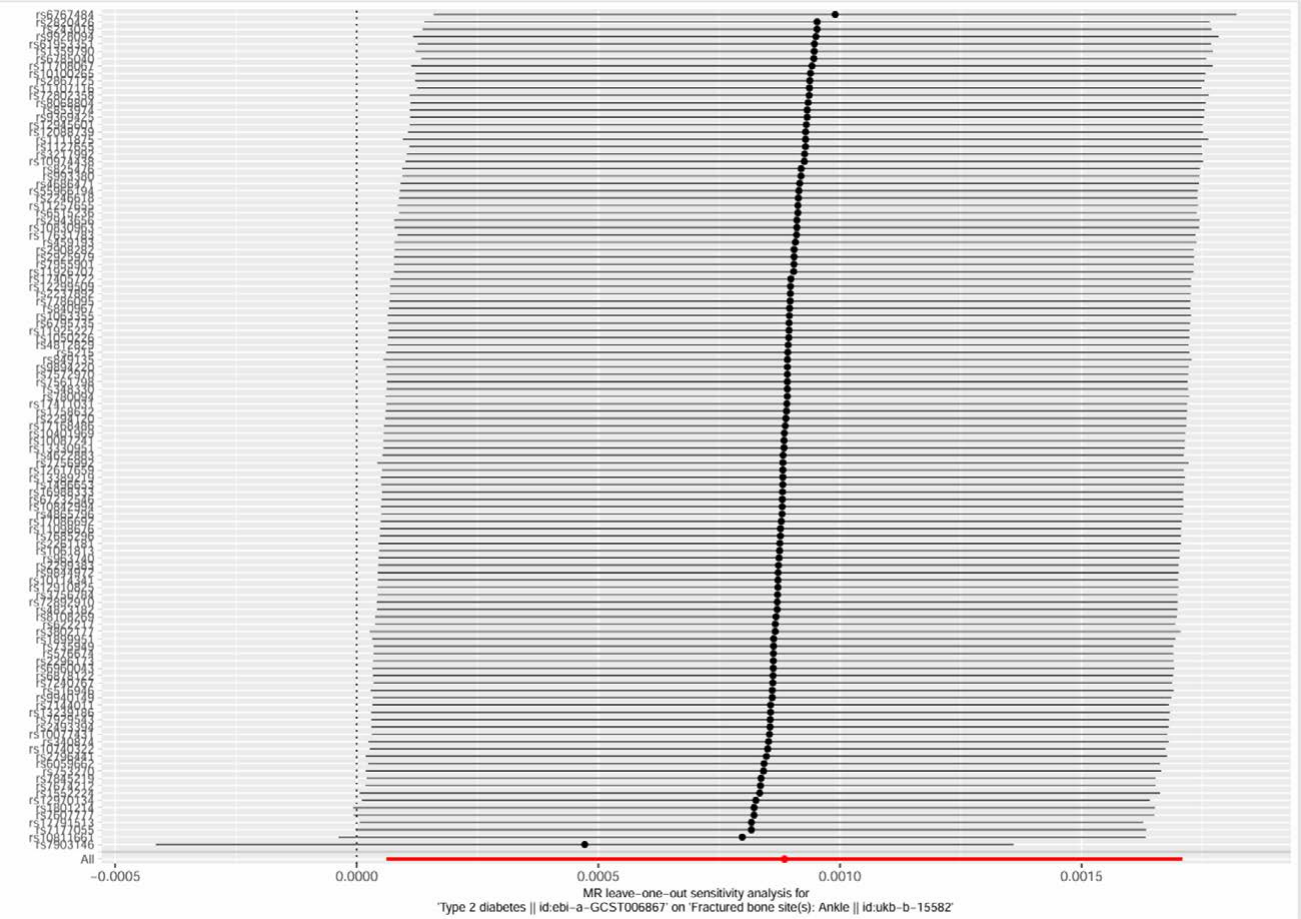
T2DM with ankle fractures by leave-one-out method. MR = Mendelian randomization, T2DM = diabetes mellitus type 2.

The results of the leave-one-out sensitivity analysis showed that, after sequentially excluding each of the 116 SNPs 1 by 1, the combined OR value of the remaining SNPs remained similar to that of the IVW method. The *P*-values were >.05, indicating that no single SNP had a significant impact on the overall results (see Fig. 3). This indicates that the effect OR values obtained by the IVW method are stable, and the results of the MR analysis are not dominated by a single SNP (the data analysis was completed by Wenren Wu and reviewed by Zhiqing Chen).

## 4. Discussion

We used large-scale GWAS pooled statistics to perform 2-sample MR analyses of 61,714 patients with T2DM and 1178 controls versus 6479 patients with ankle fractures and 453,861 controls to explore putative causal associations between T2DM and the risk of ankle fractures. The causal relationship was further confirmed by IVW, MR-Egger regression analysis, WME method, SM and weighted multivariate analysis. The results of leave-one-out sensitivity analysis showed that the results were not caused by the abnormal influence of single SNP, which enhanced the reliability of the conclusions. Based on this study, we found a positive causal association between genetically predicted T2DM and risk for ankle fractures.

This 2-sample MR Randomized study is of great clinical significance. Firstly, as the first MR study to use T2DM as exposure to estimate its causal effect on risk for ankle fractures. A great strength of this study is its 2-sample MR design, preventing the influence of potential confounding factors. Secondly, the sample of this study comes from the GWAS database,^[16]^ which makes the study an effective causal inference with high statistical power. Third, the results were extremely robust through rigorous quality control conditions and a series of sensitivity analyses. After removing SNPS associated with potential confounders, MR studies showed a causal relationship between genetically predicted T2DM and risk for ankle fractures, with an independent association between the 2.

Reduced bone turnover has been reported in T2DM, which in turn is associated with osteoporosis and fracture risk.^[17]^ Indeed, Leanza et al found that gene expression of sclerostin, a potent inhibitor of the classical Wnt signaling pathway that regulates bone homeostasis, is upregulated in T2DM.^[18]^ In addition, both hyperinsulinemia in T2DM and the aforementioned higher sclerostin levels can stimulate bone marrow lipogenesis, leading to increased bone marrow adipose tissue and senescence of bone marrow stromal cells.^[19,20]^ In turn, this leads to reduced bone quality.^[20,21]^ The long-term hyperglycemic state associated with T2D has been reported to be an accelerator for the formation of advanced glycation end products (AGEs).^[22]^ AGEs have many negative effects on bone and bone turnover; firstly, AGEs can interfere with normal osteoblast function,^[23]^ impair osteoblast development,^[22]^ and inhibit the process of osteoclast differentiation.^[24]^ In addition, AGEs can cause nonenzymatic cross-linking of type 1 collagen, thereby reducing the strength of the bone matrix.^[25,26]^ However, the study by Axelsson et al showed that in the absence of certain risk factors (such as long duration of diabetes or insulin treatment), individuals with T2DM had a lower risk of fracture compared with controls.^[27]^ Wallander et al found a significant increase in fracture risk only in individuals with T2DM using insulin compared to individuals without T2DM.^[28]^ In patients with T2DM taking oral antidiabetic drugs, fracture risk was found to be similar to the risk in individuals without T2DM. In addition, those with T2DM who did not use any medications had a lower risk of hip fracture. Sarodnik et al reported that the crude incidence of all fractures in newly treated T2DM patients was slightly lower, but certainly not higher, than in an age - and sex-matched control population.^[29]^ According to statistics, among all patients who have received ankle fracture fixation, approximately 13% are diabetic patients and 2% have diabetic complications. Nonsurgical treatment of ankle fractures in patients with diabetes may lead to a higher incidence of complications and more serious complications. Therefore, further research is still needed on how to treat patients with ankle fractures combined with type 2 diabetes.^[30,31]^

However, this study has certain limitations. First, the SNPs in the study were mainly from European populations, so our causal estimates may not be fully generalizable to other populations. Secondly, as previous studies have evaluated the causal relationship between weight, BMI and other factors and T2DM, as well as their causal relationship with ankle fractures, it was found to be causal with both ankle fractures and T2DM risk.^[32,33]^ Based on this, we did not conduct the study again. Third, the data statistical processing method of this study is single. Multiple methods should be compared for analysis. Finally, because of the lack of information, the outcome was not stratified according to the different clinical classification of ankle fractures, which is very important in clinical practice. Further studies of ankle fractures with different clinical types are needed to fully explore the association betweenankle fractures and T2DM.

## 5. Conclusion

In conclusion, this study explored the positive causal relationship between EC exposure and the outcome of BC, but the mechanism and details need to be further explored, so that clinical workers can strengthen ankle fractures screening for T2DM patients, so as to achieve early detection, early diagnosis and early treatment.

## Author contributions

**Conceptualization:** Wenren Wu, Zhiqing Chen, Binshan Zhang.

**Data curation:** Wenren Wu, Zhiqing Chen, Linfeng Luo.

**Formal analysis:** Wenren Wu, Zhiqing Chen.

**Methodology:** Wenren Wu, Binshan Zhang.

**Project administration:** Wenren Wu, Zhiqing Chen, Binshan Zhang, Linfeng Luo.

**Writing – original draft:** Wenren Wu, Zhiqing Chen.

**Writing – review & editing:** Wenren Wu, Binshan Zhang, Linfeng Luo.

**Funding acquisition:** Linfeng Luo.

## Supplementary Material


